# A Crown Whose Band Remains Invisible

**Published:** 1907-06-15

**Authors:** S. D. Ruggles

**Affiliations:** Portsmouth, O.


### CLINICS AT THE OHIO STATE MEETING.

### A Crown Whose Band Remains Invisible.

S. D. RUGGLES, D. D. S., PORTSMOUTH, O.

Prepare the root as for a Logan crown, then countersink one-sixteenth of an inch into its face well toward the lingual, a Walker-Younger trephine of a size to suit the case; that is, one that will not come so near the periphery as to endanger its strength. Now place in this groove a ring that corresponds in diameter to the trephine used, and grind it even with the face of the root. These rings are made of thirty-gauge gold or platinum, depending upon whether it is to be a porcelain or Richmond crown. (A stock of the different sizes can be made and kept on hand. The trephines used most are numbers two, three and four, and can be bought from the S. S. White Co., at one dollar each.) Leaving the ring in position, you next select a piece of metal of same thickness and of size sufficient to cover the entire surface of the root. Burnish to place, being careful to anneal frequently. Now remove the ring, place in position upon this disk and solder. Replace the ring in the grove and burnish and trim the metal disk until it corresponds to size and shape of root. Should you care to make a banded Logan, puncture the disk and ream out the root canal to fit pin, and proceed in the usual way, for either a solder case or for porcelain.

If a Richmond or all-porcelain is desired, puncture the metal covering, the canal, ream it out and fit an iridio-platinum pin, tack in place with sticky wax, remove and solder. Now you can proceed in the customary way.

It is good practice when using the Logan Crown regular to insert a ring or band with quick-setting cement, and grind even with the face of root, then set your crown as in other cases. This will prevent the root from splitting on account of undue lingual pressure.

The advantages of the method are no irritation to soft tissues due to an ill-fitting band, no metal visible and universal application to all porcelain or porcelain combined with metal.

